# Differential effects of purified low molecular weight Poly(I:C) in the maternal immune activation model depend on the laboratory environment

**DOI:** 10.1038/s41398-024-03014-7

**Published:** 2024-07-20

**Authors:** Katharina E. Tillmann, Ron Schaer, Flavia S. Mueller, Karin Mueller, Bernhard Voelkl, Ulrike Weber-Stadlbauer, Daniela D. Pollak

**Affiliations:** 1 Department of Neurophysiology and Neuropharmacology, Medical University of Vienna, Vienna, Austria; 2 Institute of Pharmacology and Toxicology, University of Zurich-Vetsuisse, Zurich, Switzerland; 3 Animal Welfare Division, Veterinary Public Health Institute University of Bern, Bern, Switzerland; 4 Neuroscience Center Zurich, University of Zurich and ETH, Winterthurerstrasse 190, 8057 Zurich, Switzerland

**Keywords:** Molecular neuroscience, Psychiatric disorders

## Abstract

The Poly (I:C) (polyriboinosinic-polyribocytidilic acid) paradigm of maternal immune activation (MIA) is most widely used as experimental model for the evaluation of the effects of gestational infection on the brain and behavior of the progeny. We have previously reported significant batch-to-batch variability in the effects of Poly (I:C), purchased from the same supplier (Sigma–Aldrich), on maternal and fetal immune responses and found these differences to be dependent on the relative amount of synthetic double-stranded RNA fragments in the high versus low molecular weight (LMW) range contained in the compound. We here resorted to Poly (I:C) purified for LMW dsRNA fragments to establish a MIA paradigm with increased reproducibility and enhanced standardization in an effort to refine the MIA paradigm and characterize its effect on offspring behavior. We found that the parallel application of LMW Poly (I:C) in two different MIA-experienced laboratories (Vienna and Zurich) yielded differential outcomes in terms of maternal immune responses and behavioral phenotypes in the offspring generation. In both experimental sites, administration of LMW Poly (I:C) induced a significant sickness response and cytokine induction in the pregnant dam and fetal brains, while the expected deficit in sociability as one main behavioral outcome parameter in the MIA progeny, was only present in the Zurich, but not the Vienna cohort. We conclude that although using Poly (I:C) purified for a defined molecular weight range reduces batch-to-batch variability, it does not make the MIA model more reliable and robust. The differential response in behavioral phenotypes of the MIA offspring between the two laboratories illustrates the highly complex interaction between prenatal and postnatal milieus - including the laboratory environment - that determine offspring phenotypic outcomes after MIA. Consequently, establishing a new MIA protocol or implementing the MIA model firstly under new or changed environmental conditions must include the assessment of offspring behavior to ensure solid and reproducible experimental outcomes.

## Introduction

In the past decades, animal models of maternal immune activation (MIA) have allowed considerable progress in our understanding of the involvement of gestational infection in the modulation of fetal brain development and in shaping neurobehavioral outcomes in the progeny [1–3]. Epidemiological findings have repeatedly implicated maternal infection during pregnancy in an increased risk for offspring neurodevelopmental disorders, including schizophrenia, autism spectrum disorders, and depressive disorders [4–6]. The establishment of reliable rodent models enabled determining the underlying molecular mechanisms and cellular pathways and to derive causality between immune stimulation of the maternal system and deviation of offspring neurodevelopment and its behavioral repercussions [7–13]. One of the most widely used substances to experimentally induce activation of the maternal immune system and to study its effect independently of the immunogenic agent itself is Poly (I:C) (polyriboinosinic-polyribocytidilic acid). Poly (I:C) is a dsRNA analog whose administration causes a cytokine-associated viral-like acute phase response [1]. The Poly (I:C) mouse model has been most successfully used in a large number of research studies to determine the pathophysiological principles of MIA and comprehensively delineate its impact on brain function and behavior of the first, second, and even third-generation descendants [7–13]. While the Poly (I:C) model has proven to be a very useful and effective tool for MIA research, it has become clear that the effect of Poly (I:C) in the MIA model may differ significantly between experiments. Recently we have experimentally investigated the observation of substantial variability in results obtained when using different batches of Poly(I:C) supplied by the same vendor [14]. In a comparative study across laboratories, we found that the relative composition of dsRNA fragment sizes of low molecular weight (LMW, 100–200 nucleotides) and high molecular weight (1000–6000 nucleotides) determined Poly (I:C) immunogenicity and its impact on the stability of pregnancy, maternal and fetal immune responses. We concluded that using a Poly (I:C) product with dsRNA fragments purified for their molecular weight may reduce the variability of research outcomes between different MIA experiments. However, although the standardization of the molecular weight range of the Poly (I:C) product in MIA studies has been recommended to reduce variability [15], the impact of a Poly (I:C) preparation with defined molecular weight RNA fragments on offspring behavior in the MIA paradigm has remained unexplored. While previous studies [15] reported more pronounced cytokine response and sickness behavior with HMW (high molecular weight) Poly (I:C) in rats, we decided to investigate LMW Poly (I:C) only as a refinement measure, since recent studies including mice [14] showed that abortion rates are considerably lower using LMW Poly (I:C) compared to HMW Poly (I:C). Here, we set out to establish a MIA protocol using a LMW Poly (I:C) product in parallel in two laboratories with ample experience in behavioral phenotyping this model. We tested two different Poly (I:C) dosages, validated maternal sickness, cytokine response in the maternal and fetal systems, and characterized the behavioral consequences in adult female and male offspring [14, 15].

## Materials and methods

### Animals

C57Bl6/N male and female mice (Charles River, Sulzfeld, Germany) 10–12 weeks were used for experimental breeding (see Table 1 for detailed information about animal numbers). Upon arrival from the vendor, all mice were housed in groups of 2–5 in individually ventilated cages in a specific-pathogen-free (SPF) holding room, which was temperature and humidity-controlled (21 ± 3°, 50 ± 10%) with a 12 h: 12 h light-dark cycle (Lights on 08:00 AM–08:00 PM (Vienna) and 09:00 PM–09.00 AM (Zurich)). All animals had *ad libitum* access to food (Ssniff Spezialdiäten, Germany (Vienna) and Kliba AG, Switzerland (Zurich)) and water throughout the entire study. All animal experiments were conducted in agreement with the EU Directive 2010/63/EU for animal experiments and approved by the Austrian (License number: BMBWF- 2021-0.150.413) and Swiss authorities (License number: ZH124/2020). Reporting of the animal experiments is in accordance with the ARRIVE guidelines. All efforts were made to minimize the number of animals used and their burden.

**Table 1:** Overview of numbers of animals used, total and separated per lab and group.

**Maternal plasma and fetal brains**^**a**^		**# of dams paired**	**# of dams injected**				**# of dams measured**^**b**^				**# living offspring**								**Total per experiment**
			PIC 10 mg	PIC 20 mg	CON		PIC 10 mg	PIC 20 mg	CON										**dams measured**
**VIE/ZHR**	VIE	80	9	10	14		9	10	9										**28**
	ZHR	50	12	12	11		9	11	10										**30**
**Behavior**		**# of dams paired**	**# of dams injected**				**# of litters**				**# Offspring**								**Total number of dams injected and offspring tested**
			PIC		CON		PIC		CON		PIC				CON				
											♀		♂		♀		♂		
**VIE/ZHR 10 mg**	VIE	100	16		18		10		13		38		41		39		59		**211**
	ZHR	40	12		12		10		11		29		30		30		30		**143**
**VIE 20 mg**	VIE	60	10		12		9		8		30		27		27		33		**139**
**VIE Reinfection**	VIE	36	PIC/PIC	PIC/CON	CON/PIC	CON/CON	PIC/PIC	PIC/CON	CON/PIC	CON/CON	PIC/PIC		PIC/CON		CON/PIC		CON/CON		
											♀	♂	♀	♂	♀	♂	♀	♂	
			5	3	4	6	4	3	3	5	15	18	12	13	10	12	7	10	**115**
**Total per procedure**		**366**	**166**								**510**								**666**

The total number of animals used contains injected dams and living offspring, number of dams mated has to be considered separately. Bold values represent total values (sum).

^a^dams injected from these groups were used for maternal plasma as well as for fetal brains.

^b^per dam, 2 fetal brains were measured, fewer dams measured than injected is due to pregnancy losses.

### Timed mating and maternal immune activation

Experimental breedings began after two weeks of habituation to the respective mouse vivarium using a previously described timed mating procedure (Khan et al., 2014, Mueller et al., 2018, respectively). In Vienna, animals were mated for one single night only, and pregnancies were confirmed post-mating days 7–10 by the evaluation of weight gain of the dams. In Zurich, dams were mated for several consecutive nights, and a plug check was performed each morning. Once the plug check was positive, the dam was moved to a new cage, and the preceding night was considered the night of conception. The day of the single housing (Vienna) or observation of a vaginal plug (Zurich) was referred to as gestational day (GD) 0.5. Pregnant dams were randomly assigned to either Poly (I:C) treatment or treatment with endotoxin-free 0.9% NaCl (B. Braun, Switzerland) vehicle solution. For all procedures, LMW Poly (I:C) (cat.#: tlrl-picw) was obtained from Invivogen (USA). The same lot of Poly (I:C) (Lot #PIW-41-05) was used throughout the study for both laboratories.

Poly (I:C) was administered through a single intraperitoneal (i.p.) injection on GD 12.5 at 10 mg/kg or 20 mg/kg using an injection volume of 10 ml/kg. Immediately after Poly (I:C) or vehicle administration, dams were placed back into their home cages and left undisturbed until the assessment of the sickness behavior and/or the collection of maternal and fetal tissues. Offspring of Poly (I:C)- or vehicle-treated mothers were weaned on postnatal day (PND) 21 and littermates of the same sex were maintained in groups of 2–5 animals per cage. For the reinfection experiment, Female F1 offspring (LMW Poly (I:C) and vehicle) were injected with LMW Poly (I:C) or vehicle on GD 12.5 in the F1 generation resulting in four groups (PIC/PIC; PIC/CON; CON/PIC; CON/CON) for the evaluation of the F2 generation. Standardized detailed information is provided in the Reporting Guidelines Checklist in Supplementary Table 1 for Zurich and Supplementary Table 2 for Vienna.

### Assessment of sickness score

To assess signs of sickness in response to Poly (I:C) all animals were scored two hours after injection using predefined criteria [16], including body position, ptosis (drooping eyelids), piloerection, coat condition, reaction to change in environment (cage, assessed by manual interaction within the cage) and nest condition. In both laboratories, sickness scores were determined by one experimenter each, who was blinded to the experimental conditions. For every mouse and criterion, a value from 0 to 3 was assigned, with 0 indicating no signs of sickness and 3 indicating the highest degree.

### Collection of maternal plasma and fetal tissue

3 h post-injection, pregnant mice were decapitated, and trunk blood was collected in EDTA-coated tubes. Blood was kept on ice for less than. 20 min before centrifugation (10.000 rpm, 10 min, 4 °C) to collect plasma, which was stored at −20 °C until further use. Fetal brain tissues were collected as described before [17, 18]. In brief, the abdominal cavity of the dam was exposed to collect the uterus, which was placed into a petri dish filled with ice-cold PBS. Decidual tissue and yolk sac were then removed for individual fetuses, and the fetuses were further dissected to obtain fetal brain tissue according to landmarks shown in (Supplementary Fig. 1). Isolated fetal brain tissue was placed in Eppendorf tubes, snap-frozen by immersion in dry ice and stored at −80 °C. Whenever possible, fetal brain tissue from two fetuses per uterine horn was collected for each pregnant dam by selecting fetuses in the most caudal uterine position.

Fetal brains from each dam were pooled and incubated in ice-cold Roche complete lysis buffer (Complete™ Lysis-M, Sigma–Aldrich, Switzerland) for 20 min before lysing using a tissue lyser (tissue Lyser II, Quiagen®) for 3 min at a frequency of 20/s. After centrifugation (12,000 rpm at 4 °C for 20 min) the supernatants were removed and frozen and stored at −80 °C until the cytokine assays were performed.

### Quantification of cytokines

Cytokine levels in maternal plasma and fetal brains were quantified using a Meso-Scale Discovery (MSD) V-plex electrochemiluminescence assay for mice as previously described (Mueller et al., 2018, 2019; Notter et al., 2018). The panel included interleukin (IL) IL-1β, IL-6, IL-10, tumor necrosis factor (TNF)-α and interferon (IFN)-γ. V-plex 96-well plates coated with primary antibodies directed against the targeted cytokines were treated with the corresponding detecting antibodies, which were pre-labeled with SULFO-TAGTM (MSD, USA). The plates were read using the MESO QuickPlex SQ 120MM (MSD) imager and analyzed using MSD’s Discovery Workbench analyzer and software package. Plasma and fetal brain lysates were diluted 2-fold and were run in duplicates according to the manufacturer’s instructions. The detection limits were 0.11 pg/ml for IL-1β, 0.61 pg/ml for IL-6, 0.94 pg/ml for IL-10, 0.13 pg/ml for TNF-α and 0.04 pg/ml for IFN-γ.

### Behavioral testing

#### General information

Behavioral testing was initiated when offspring reached 8–12 weeks of age and included tests for sociability (Social interaction test), innate anxiety behavior and locomotor activity (Open field test) and Fear conditioning (second Vienna cohort only). These tests are widely used in animal models of MIA [5, 16, 19] and extensively validated [17, 20, 21]. The order of testing was always kept constant (1. Open field test; 2. Social interaction test; 3. Fear conditioning – where applicable) and a resting interval of at least 3 days was kept between tests.

#### Open field test

A standard open field test was applied to assess basal locomotor activity and innate anxiety-like behavior. The testing arena (40 × 40 cm) was made of white Plexiglas and surrounded by walls (35 cm in height). The testing room was maintained at fixed lighting conditions (30 lx) and a digital camera was mounted directly above the arena. Activity was monitored by a computational tracking system (Activity Monitor, MedAssociates, USA) in Vienna, and Ethovision (Noldus Information Technology, Netherlands) in Zurich, respectively. In each case, the animals were gently placed in the center of the arena and allowed to freely explore for the testing duration of 10 min. For the purpose of data collection, the arena was conceptually partitioned into two areas: a center zone measuring 15 × 15 cm^2^ in the middle of the area and a peripheral zone occupying the remaining area. The dependent measures were the total distance moved (cm) in the entire arena.

#### Social interaction test

Social interaction was determined by analyzing the relative exploration time between an unfamiliar congenic mouse and an inanimate object using a protocol established before [12, 21]. The test apparatus was made of Plexiglas and consisted of three identical arms (Vienna: 37 cm × 6 cm × 16 cm; Zurich: 50 cm × 9 cm × 10 cm; length × width × height). The three arms radiated from a centrally located equilateral triangle spaced 120° from each other. Two out of the three arms contained a rectangular stranger cage. The third arm did not contain a cage and served as the starting zone (see below). All animals were habituated to the test apparatus on the day before social interaction testing for 5 min. During the test phase on the following day, one stranger cage contained an unfamiliar C57BL6/N mouse of the same sex (10–12 weeks of age), whereas the other stranger cage contained an inanimate dummy object. This inanimate dummy object consisted of three black Lego bricks in Vienna and a black scrunchy in Zurich. The allocation of the unfamiliar live mouse and inanimate dummy object to the two stranger cages was counterbalanced across experimental groups. To start a test trial, the test mouse was gently placed in the starting arm and allowed to explore freely for 5 min. A digital camera was mounted above the test apparatus which captured and transmitted images to the EthoVision tracking system. Behavioral observations were made by an experimenter who was blinded to the experimental conditions, and social interaction was defined as nose contact within a 5-cm interaction zone. For each animal, the relative time spent with the unfamiliar mouse was calculated by the formula [(time spent with the mouse) / (time spent with the inanimate object + time spent with the mouse)] × 100.

#### Fear conditioning

In the Vienna cohort, a standard Pavlovian conditioning protocol was employed as previously reported [11]. Briefly, mice were handled by the experimenter on the two days directly preceding the start of the FC paradigm. On trial days, mice were allowed to habituate for approximately 45 min in a holding room adjacent to the experimental room. The 12 min conditioning training session consisted of the delivery of three pairings between an unconditioned stimulus (US: foot shock, 0.3 mA, 1 s) and a conditioned stimulus (CS: white noise, 75 dB, 30 s) with an inter-trial interval of 180 s. Cued recall was tested 24 h after the training session in a test trial lasting 720 s in total, with the preCS phase being 360 s long while the CS was presented for 180 s, followed by a postCS phase of 180. The response to the CS was evaluated as the freezing levels during the presentation of the CS (preCS-CS). Freezing behavior (percentage of time spent freezing, defined as lack of movement for at least 0.5 s, or 15 frames at a sampling rate of 30 frames/s) was automatically recorded using the ‘NIR Video Fear Conditioning Contextual Package for Mouse’ system and accompanying Video Freeze software (Med Associates, USA).

#### Statistics

Statistical analysis was performed in the R statistical programming environment [22]. For comparing treatment effects on cytokine concentration in maternal plasma, two-way type 1 ANOVAs with lab and treatment and the lab-by-treatment interaction were used. Separate tests were run for cohorts of dams receiving 10 and 20 mg/kg Poly (I:C). Behavioral measures and sickness scores were compared with three-way or two-way type 1 ANOVAs with lab, treatment, and sex (not for sickness score) and all interaction terms as fixed factors. After finding a significant interaction effect of lab and treatment for social interaction, post-hoc two-way ANOVAs with treatment and sex and the sex-by-treatment interaction were done for each lab. Additional mixed effect models using the package lme4 [23] including dam as random factor showed that dam explained only a negligible proportion of the overall variance and its inclusion did not affect outcomes for the main factors. We identified multivariate outliers for the behavioral measures using the package mvoutlier. Re-calculating ANOVA statistics after the removal of these outliers did not change the outcomes for treatment, lab, and lab-by-treatment interaction, corroborating the robustness of the results. For investigating treatment effects in a new cohort of dams receiving 20 mg/kg at the lab in Vienna, we used two-way type 1 ANOVAs with treatment, sex, and sex-by-treatment interaction as fixed factors. Re-calculating ANOVA statistics after the removal of multivariate outliers did, again, not change the outcome for treatment effects. For analyzing treatment effects in the F2 generation after reinfection, we used three-way type 1 ANOVAs with the treatment of dams in F0 and F1, sex, and all interaction terms as fixed factors. Re-calculating ANOVA statistics after the removal of multivariate outliers did not change the outcome for treatment effects. For comparing treatment effects on cytokine concentration in fetal brains, two-way type 1 ANOVAs with lab and treatment and the lab-by-treatment interaction were used. Separate tests were run for cohorts of dams receiving 10 and 20 mg/kg. Assumptions for ANOVA were checked by visually inspecting QQ-plots and scale location plots using the package lindia [24]. QQ-plots showed that residual distributions for cytokines in dam plasma and brain tissue were partly heavy-tailed but symmetrical. Residuals for social interaction were slightly left-skewed and light-tailed, though given that the observed residuals fitted the prediction line well over a wide range, data transformation was not considered necessary.

## Results

### Maternal behavioral and fetal cytokine responses upon LMW Poly (I:C) challenge

**Fig. 1:**
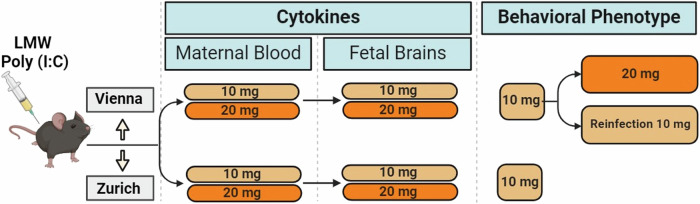
Schematic depiction of the experimental timeline. The establishment of the LMW Poly (I:C) maternal immune activation (MIA) paradigm comprised the evaluation of maternal sickness behavior, cytokine responses in the maternal plasma and fetal brains using 10 and 20 mg/kg Poly (I:C) dosages. Behavioral phenotyping was conducted using 10 mg/kg in the Vienna and Zurich sites. In Vienna a second cohort was used to test the consequences of Poly (I:C) at 20 mg/kg on offspring behavior. In a third Vienna cohort (“reinfection”), pregnant MIA and control offspring (F1) were treated with 10 mg/kg Poly (I:C) or saline vehicle, and adult offspring were behaviorally phenotyped.

**Fig. 2:**
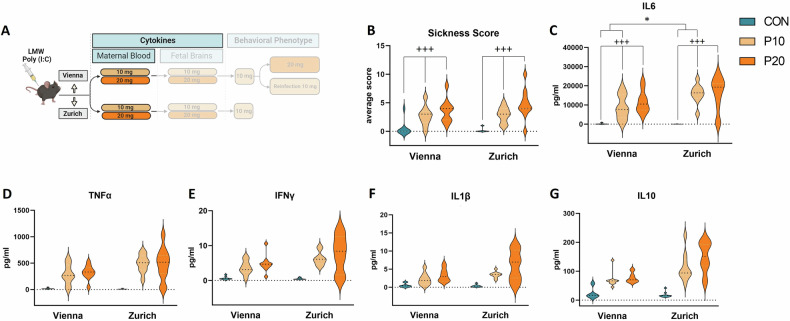
Maternal sickness behavior and plasma cytokine responses in the LMW Poly (I:C) MIA protocol. **A** Schematic depiction of the experimental design. **B**–**G** Significant effects of Poly (I:C) treatment for both dosages (10 mg/kg and 20 mg/kg) were observed for all parameters tested: **B** maternal sickness behavior, maternal plasma levels of **C** IL-6, **D** TNF-α, **E** IFN-γ, **F** IL-1β, and **G** IL-10. All data are presented as violin plots, with dotted lines showing median, n = 8–10 animals/group; significant main lab (*p < 0.05, **p < 0.01, ***p < 0.001) and treatment (+p < 0.05, ++p < 0.01, +++p < 0.001) effects are indicated.

As a first step in the establishment of the LMW Poly (I:C) MIA model (Fig. 1) we set out to determine the immediate immune response of the pregnant female to the Poly (I:C) challenge at GD 12.5 (Fig. 2A). To this end we monitored sickness behavior of the dam and evaluated maternal plasma levels of a panel of cytokines previously characterized in our and others´ MIA studies (eg [14, 25–27]). The selected cytokines comprised IL-1β, IL-6, IL-10, TNF-α, and IFN-γ, which have not only been found dysregulated in the MIA paradigm, but also form part of the inflammatory profiles in the neurodevelopmental disorders reflected in the MIA model (eg [28–31]). Three groups (10 mg/kg or 20 mg/kg Poly (I:C) and vehicle controls) of animals were included in both labs (Vienna and Zurich). We focused on comparisons of either dosage (10 mg/kg or 20 mg/kg) Poly (I:C) to controls (treatment effect), while also monitoring possible differences between the Vienna and the Zurich sites (lab effect) and interactions between the above (lab x treatment interactions). For sickness behavior we found a significant treatment effect for the 10 and 20 mg/kg dosage (F_(1,33)_ = 33.758, p < 0.001; F_(1,36)_ = 47.153, p < 0.001, respectively, Fig. 2B). Cytokine analysis revealed highly significant treatment effects for all parameters tested (IL-6, TNF-α, IFN-γ, IL-1β, IL-10) for both dosages of Poly (I:C)(10 mg: IL-6) (F_(1,33)_ = 88.359, p < 0.001), TNF-α (F_(1,33)_ = 86.434, p < 0.001), IFN-γ (F_(1,33)_ = 91.698, p < 0.001), IL-1β (F_(1,33)_ = 57.683, p < 0.001), IL-10 (F_(1,33)_ = 54.602, p < 0.001); 20 mg: IL-6 (F_(1,36)_ = 63.535, p < 0.001), TNF-α (F_(1,36)_ = 51.799, p < 0.001), IFN-γ (F_(1,36)_ = 35.905, p < 0.001), IL-1β (F_(1,36)_ = 33,197, p < 0.001), IL-10 (F_(1,36)_ = 93.540, p < 0.001). At 10 mg/kg we additionally observed lab effects for IL-6 (F_(1,33)_ = 5.049, p = 0.031), TNF-α (F_(1,33)_ = 4.448, p = 0.043), IFN-γ (F_(1,33)_ = 5.349, p = 0.027) as well as lab x treatment effects for IL-6 (F_(1,33)_ = 7.230, p = 0.011), TNF-α (F_(1,33)_ = 6.936, p = 0.013), IFN-γ (F_(1,33)_ = 8.836, p = 0.005), IL-1β (F_(1,33)_ = 4.930, p = 0.033) and IL-10 (F_(1,33)_ = 6.611, p = 0.015). At 20 mg/kg there were only significant effects for IL-10 (main effect of lab (F_(1,36)_ = 12.342, p = 0.001) and lab: treatment interaction (F_(1,36)_ = 16.032, p < 0.001)) (Fig. 2C–G). These data demonstrated that treatment with either dosage of LMW Poly (I:C) at GD 12.5 elicited sickness behavior and a strong and robust circulating cytokine response in pregnant dams in both research sites with some lab-specific differences. A summary of all statistical findings is reported in Supplementary Table 3A, B.

**Fig. 3:**
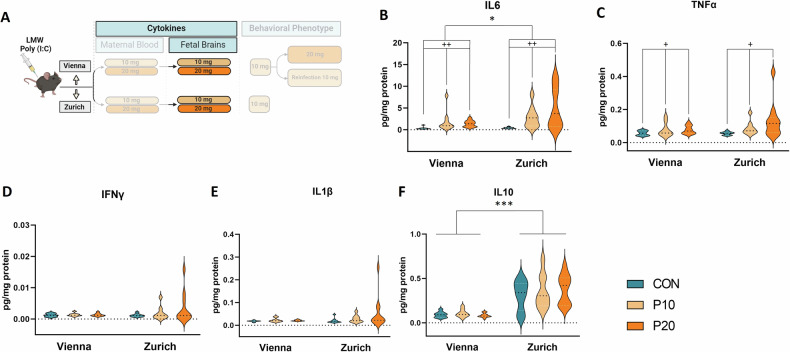
Cytokine levels in fetal brains after maternal treatment with LMW Poly (I:C). **A** Schematic depiction of the experimental design. **B**–**F** Depiction of cytokine levels in fetal brains upon maternal treatment with LMW Poly (I:C) (10 mg/kg and 20 mg/kg). **B** IL-6, **C** TNF-α, **D** Interferon-gamma, **E** IL-1β and **F** IL-10. All data are presented as violin plots, with dotted lines showing median, n = 9–11 animals/ group; significant main lab (*p < 0.05, **p < 0.01, ***p < 0.001) and treatment (+p < 0.05, ++p < 0.01, +++p < 0.001) effects are indicated.

We next examined the cytokine response in fetal brains (Fig. 3A). We tested the same cytokine panel as in the maternal plasma using both 10 mg/kg and 20 mg/kg of Poly (I:C). For IL-6 and TNF-α we found significant main effects of treatment for both dosages (10 mg/ kg: F_(1,33)_ = 12.772, p = 0.001; F_(1,33)_ = 6.562, p = 0.015; 20 mg/ kg: F_(1,36)_ = 12.888, p < 0.001; F_(1,36)_ = 6.862, p = 0.013) (Fig. 3B, C). Significant lab effects were revealed for IL-10 (10 mg/ kg: F_(1,33)_ = 29.545, p < 0.001; 20 mg/ kg: F_(1,36)_ = 44.433, p < 0.001) and for IL-6 (20 mg/ kg: F_(1,36)_ = 5.469, p = 0.025). Additionally, we observed a lab x treatment effect for the expression of IL-6 in fetal brains of mothers treated with 20 mg/kg (F_(1,36)_ = 4.733, p < 0.036). Levels of IFN-γ and IL-1β in fetal brains were unaffected by Poly (I:C) treatment of either dosage in either lab (Fig. 3D–F). This observation suggests a potential impact of the laboratory environment on fetal immune responses upon Poly (I:C) challenge of the mother.

### Between-laboratory variability in the behavioral phenotype of adult MIA offspring

**Fig. 4:**
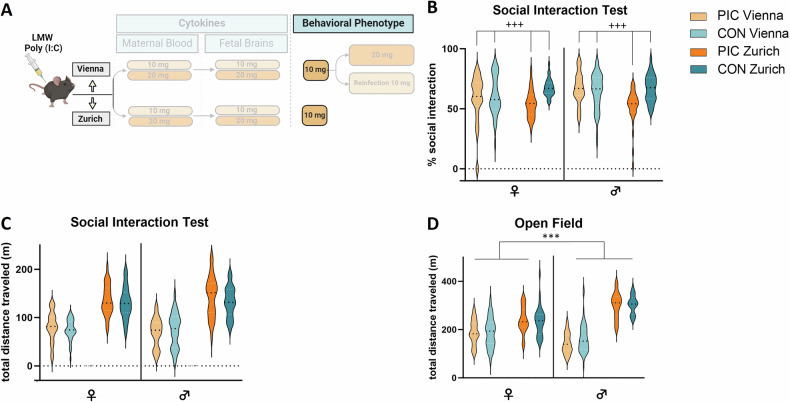
Behavioral phenotype of adult MIA offspring after maternal treatment with 10 mg/kg LMW Poly (I:C). **A** Schematic depiction of the experimental design. **B** Sociability (percentage of time engaged in social interaction), **C** total distance traveled (during social interaction task) and **D** exploratory activity (total distance traveled in the Open field) in male and female offspring of the Vienna and Zurich cohorts. All data are presented as violin plots, with dotted lines showing the median, Vienna: n = 32–58 animals/group, Zurich: n = 29–30 animals/group; significant main lab (*p < 0.05, **p < 0.01, ***p < 0.001) and treatment (+p < 0.05, ++p < 0.01, +++p < 0.001) effects are indicated.

Given the expected immunogenic effect on the pregnant dams and significant response in the fetal brains with both dosages of LMW Poly (I:C), we went on to evaluate offspring behavioral phenotype using the lower dosage (10 mg/ kg) (Fig. 4A). We used social interaction as a main outcome parameter, as we had previously found a robust reduction of the preference to interact with an alive mouse over an inanimate object in MIA offspring [7, 10, 12]. Total distance moved in the Open field was used as control procedure to exclude unspecific effects of MIA on exploratory activity as in earlier studies [7–13]. However, while a significant effect of Poly (I:C) treatment on social interaction was revealed (F_(1,263)_ = 15.161, p < 0.001) as represented in a reduced percentage of time interacting with the stranger mouse, we also observed a significant interaction between laboratory and treatment (F_(1,263)_ = 15.072, p < 0.001). Post-hoc tests showed that the expected social interaction deficit in MIA offspring was only caused by differences in the Zurich cohort (F_(1,115)_ = 47.038, p < 0.001) but not the Vienna cohort (F_(1,148)_ = 0.105, p = 0.746, Fig. 4B). Additionally, we found significant lab effects as well as lab x sex effects for distance traveled (F_(1,288)_ = 21.437, p < 0.001; F_(1,288)_ = 18.833, p < 0.001, respectively). No differences between MIA and control animals for total difference traveled in the Open Field were found (Fig. 4C, D), indicating that alterations in activity could confound the readouts of the social interaction test. As such, these data show that the interaction with the specific laboratory environment may modulate the effect of Poly (I:C) treatment on offspring behavior, despite detectable cytokine responses to Poly (I:C) administration in the maternal and the fetal compartments.

**Fig. 5:**
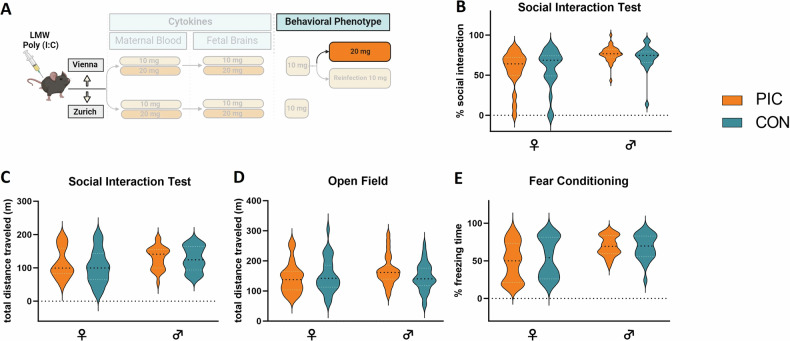
Behavioral phenotype of adult MIA offspring after maternal treatment with 20 mg/kg LMW Poly (I:C) in the Vienna cohort. **A** Schematic depiction of the experimental design. **B** Sociability (percentage of time engaged in social interaction), **C** total distance traveled (during social interaction task), **D** exploratory activity (total distance traveled in the Open field) and **E** learned fear (percentage of time freezing in response to presentation of the conditioned stimulus) in male and female offspring of the Vienna cohort. All data are presented as violin plots, with dotted lines showing median, n = 21–33 animals/group; no significant main treatment effects were detected, significant main sex effects are not indicated.

Since no consequences of Poly (I:C) challenge for offspring behavioral phenotype were revealed in Vienna using 10 mg/kg, we next explored whether administration of the higher dosage (20 mg/kg) would result in the anticipated deficit in the MIA progeny in the Vienna cohort (Fig. 5A). This was motivated by the consideration that the increase in cytokine levels in the Vienna cohort, which was partly less pronounced than in Zurich, might not have been sufficient to elicit a behavioral phenotype in the progeny. However, no differences in social interaction between MIA and control female and male offspring were observed (Fig. 5B), and no effect of Poly (I:C) treatment on distance traveled in the Open field (Figs. 5C, D). We then explored the possibility that an offspring phenotype in response to maternal Poly (I:C) treatment at 20 mg/kg offspring would become overt in a different behavioral paradigm and assessed conditioned fear, which was previously demonstrated to be altered in MIA progeny [12], but found no effect in the cued fear response (Fig. 5E). Main effects of sex were found in the Social interaction and Fear conditioning tests (F_(1,102)_ = 26.103, p < 0.001; F_(1,102)_ = 18.839, p < 0.001, respectively). Hence, the lack of a behavioral phenotype in MIA offspring in Vienna was not dependent on the Poly (I:C) dosage, but may rather be influenced by other parameters of the prenatal and/or postnatal environment.

### Exposure of pregnant F1 female MIA offspring to Poly (I:C) (“reinfection”) does not lead to behavioral alterations in the F2 generation

**Fig. 6:**
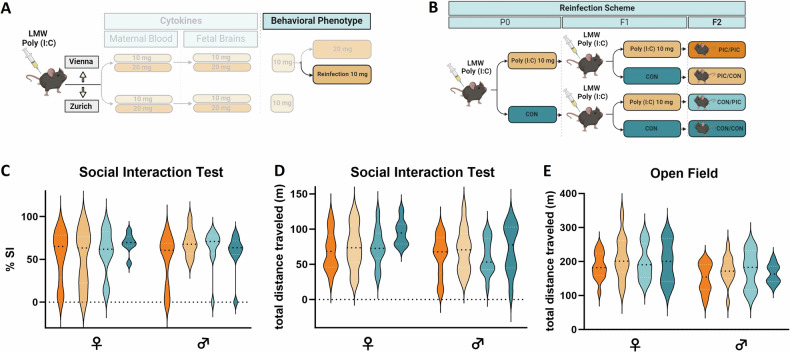
Behavioral characterization of second-generation MIA offspring after gestational Poly (I:C) challenge of F1 MIA offspring in the Vienna cohort. **A** Schematic depiction of the experimental design. **B** Treatment scheme for the “reinfection” paradigm to test the effect of gestational immune challenge of pregnant F1 MIA offspring on F2 behavioral phenotypes. **C** Sociability (percentage of time engaged in social interaction), **D** total distance traveled (during social interaction task) and **E** Exploratory activity (total distance traveled in the Open field) in male and female F2 generation offspring of F1 MIA (PIC) or control (CON) mothers with a history of MIA or control treatment (PIC/PIC, PIC/CON, CON/PIC, PIC/PIC). All data are presented as violin plots, with dotted lines showing the median, n = 17–33 animals/group; no significant main effects were detected.

We have previously found the effect of MIA on offspring behavioral phenotypes, including deficient maternal care behavior, augmented depression-like behavior, reduced sociability, and increased fear memory to extend to subsequent generations [11, 12]. In this context, we have found both, persistence and modification of behavioral traits in the MIA progeny [11, 12]. We thus hypothesized that an effect of Poly (I:C) might be latent in the F1 generation in the Vienna cohort but could be unmasked in the F2 generation after a possible exacerbation by F1 female MIA re-exposure to the same immune challenge during their own pregnancy (“reinfection”) (Fig. 6A). To experimentally address this possibility, we generated F1 MIA offspring after maternal exposure to 10 mg/kg Poly (I:C) alongside respective vehicle controls (F0 generation). F1 females (MIA and controls) were divided into two groups, each exposed to either Poly (I:C) (10 mg/kg) or vehicle at GD 12.5, thereby generating four groups of F2 offspring (Fig. 6B). Adult female and male offspring born to these mothers (F2 generation) were behaviorally examined (Fig. 6B). Social interaction and performance in the Open Field of F2 offspring were unaffected by Poly (I:C) treatment of either the P0 or the F1 generation dams (Fig. 6C–E). Collectively these results demonstrate that in the Vienna lab adult MIA F1 and F2 offspring do not express the expected behavioral abnormalities previously observed in the MIA Poly (I:C) model and that “reinfection” of F1 female offspring does not alter the behavior of their offspring under these conditions.

## Discussion

Purifying Poly (I:C) for dsRNA fragments of a designated molecular weight range has been suggested to reduce experimental variability and support the reproducibility of experimental results [14]. Here we sought to establish the Poly (I:C) MIA model using a LMW product with a focus on defining offspring the behavioral phenotype most commonly assessed in studies in the basic and translational psychiatry research fields. We used the opportunity of setting up a new MIA protocol using LMW Poly (I:C) by Invivogen to monitor experimental outcomes in two laboratories in parallel, to explore whether reducing batch-to-batch variability in the immunogenicity of the Poly (I:C) compound would also contribute to lessening differences obtained between experimental sites using the same Poly (I:C) product.

The phenotypic observation of the mothers (sickness behavior) and the quantification of proinflammatory cytokines in response to the LMW Poly (I:C) stimulation showed a robust treatment response for both dosages which was also reflected in immune activation in the fetal brains as a prerequisite for inducing behavioral alterations in the MIA progeny. Yet, the observed differences in the levels of individual cytokines between labs, either as main effect or dependent on treatment/ dosage, already indicate that the immune response in the model is significantly influenced by the specific animal husbandry and its condition. The current study does not permit identifying if and which of those distinctions contribute to the resulting offspring phenotypic variability between the two experiments sites. While several behavioral phenotypes in the adult offspring, including depression-like behavior, reduced sociability, and alterations in fear learning have been reported in the MIA paradigm, we here decided to focus on social behavior as a main outcome parameter, considering social disconnection/ impaired sociability as a shared symptom of several mental disorders for which the Poly (I:C) paradigm is used as a model in translational psychiatry research. Moreover, a reduction in social interaction has been robustly observed in earlier studies and across species [7, 10, 12, 32, 33]. However, while 10 mg/ kg LMW Poly (I:C) at GD 12.5 led to the expected deficit in social interaction in Zurich, no effect was apparent in the Vienna laboratory, despite comparable maternal sickness levels and significant cytokine induction in the maternal plasma and fetal brains in both laboratory environments. The lack of a behavioral phenotype in the Vienna lab even after increasing the Poly (I:C) dosage suggests that rather than only the concentration of the compound and the subsequently produced immune response, other pre- and/or post-natal factors may be determining the effects of MIA on offspring behavior.

Notably, while a variety of factors were harmonized across both research sites, including testing protocols for all behavioral paradigms, some differences between laboratories persisted. These include e.g. the caging system, which can indeed affect cytokine- and chemokine responses, as well behavioral outcomes in the MIA model [21], the light schedule in the facility (the Zurich lab uses a reversed light-dark cycle), animal food and consequently the microbiome as well as different approaches for generating timed pregnancies in the two sites. However, these slight variations reflect the typical differences in experimental conditions between research sites, which can and will never be 100% identical between independent studies. Our data thus indicates that using maternal sickness behavior or levels of selected individual cytokines as a proxy in the establishment of the Poly (I:C) MIA protocol may be not sufficient for predicting offspring behavioral phenotypes.

Both laboratories have independently observed that the effect of MIA on offspring phenotype manifested not only in the first, but also the second and even third generation, including both persistence and modifications of behavioral traits across generations [11, 12, 34]. Given the highly significant increase of maternal cytokines paired with a lack of offspring phenotype with the lower dose in Vienna, we tested the possibility that the behavioral effect was latent in the F1 offspring but could be unmasked by priming MIA offspring with an additional MIA stimulus and testing F2 generation offspring. However, we observed no effect of MIA on offspring behavior in any of the experimental groups, although we cannot rule out that other “second hits”, such as stress experience or exposure to psychostimulant drugs may uncover or jointly elicit a behavioral phenotype under these conditions.

While in both laboratory environments, LMW Poly (I:C) elicits a robust immune activation in the maternal and fetal systems, the results concerning offspring behavior differ substantially. These observations jointly suggest that the immune response itself and the interaction with other factors form an intricate web of interrelationships that determine long-term outcomes in the MIA model. Focusing on a specific set of cytokines when investigating the immunogenic effects of LMW Poly(I:C), as done here as well as in earlier studies [14], bears the risk that differences in other cytokines, which could potentially underlie the variations in offspring phenotypes, may not be detected. Indeed, other cytokines and molecular elements downstream of the signaling cascades activated by Poly (I:C), many of which remain to be discovered, may interact with distinct determinants of the specific laboratory setting [14]. Such factors could include, but are not limited to e.g., the maternal microbiome, physical variables within the housing and testing environment (air pressure and flow, humidity) or postnatal influences within the offspring social groups or influences of experimenters and staff. These may then also not only directly affect the developing fetus, but also indirectly modulate other relevant variables in the model, such as maternal care behavior [13], which remains to be tested using LMW Poly (I:C).

Against this background, our study clearly demonstrates that using maternal sickness behavior or levels of selected individual cytokines as a proxy in the establishment of the Poly (I:C) MIA protocol may be not sufficient for predicting offspring behavioral phenotypes. While using Poly (I:C) compounds with dsRNA fragments purified for their molecular weight may reduce batch-to-batch variability, the environmental conditions specific and intrinsic to each laboratory importantly determine offspring behavioral outcomes for one of the most commonly used models in translational psychiatry. As such, the successful implementation of a MIA model in each specific laboratory warrants titrating and defining the optimal settings and needs to extend beyond the assessment of maternal sickness and/or cytokine responses. In this regard, whenever establishing a MIA model or when introducing changes to protocols, the animal husbandry or the laboratory environment, a pilot experiment that includes not only the assessment of prenatal cytokine profiles, but also the evaluation of offspring behavioral phenotypes, is highly recommended. Verifying offspring phenotype in a small-scale pilot approach is also favorable in consideration of the 3 R policy, as it will prevent larger cohorts of animals being used with possibly unsatisfying experimental outcomes.

### Supplementary information

Supplementary Figure 1 Legend

Supplementary Figure 1

Supplementary Table 1

Supplementary Table 2

Supplementary Table 3

Supplementary Figure 2

Supplementary Figure 2 Legend

## Data Availability

The data that support the findings of this study are available upon reasonable request from the corresponding author.
