# Supplementary material for: Differential effects of purified low molecular weight Poly(I:C) in the maternal immune activation model depend on the laboratory environment
Source: Transl Psychiatry. 2024 Jul 20;14:300. doi: 10.1038/s41398-024-03014-7 (PMC11271296; doi:10.1038/s41398-024-03014-7)
Supplement: Supplementary file 1 — Supplementary Figure 1 Legend [file 41398_2024_3014_MOESM1_ESM.pdf]

**Supplementary Figure 1.** Photomicrograph of a representative fetus collected on gestation day (GD) 12 in C57BL6/N mice. The dashed red line indicates the incision line used to separate the main part of the fetal brain from the rest of the body. The fetal brain tissue was then further processed to measure cytokine and chemokine contents as described in the main manuscript.
