# Supplementary figures and images for: Differential effects of purified low molecular weight Poly(I:C) in the maternal immune activation model depend on the laboratory environment
Source: Transl Psychiatry. 2024 Jul 20;14:300. doi: 10.1038/s41398-024-03014-7 (PMC11271296; doi:10.1038/s41398-024-03014-7)

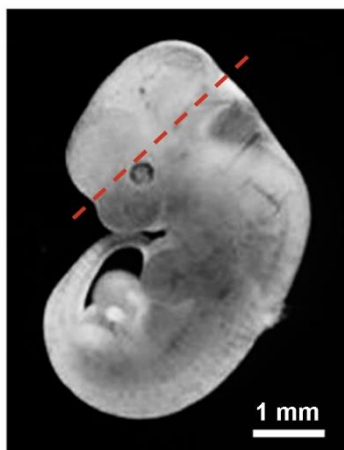

*Supplementary Figure 1*

Supplement: Supplementary file 2 — Supplementary Figure 1 [file 41398_2024_3014_MOESM2_ESM.pdf]

**A**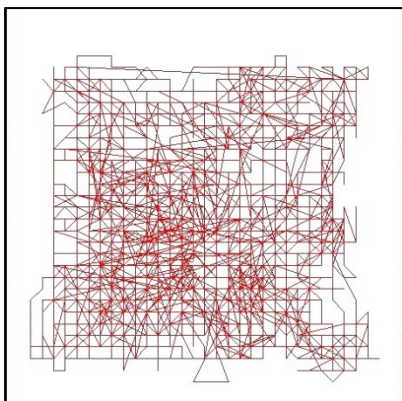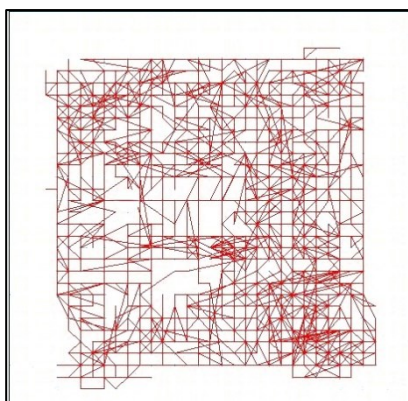**B**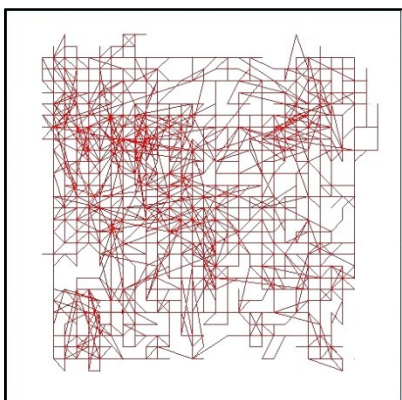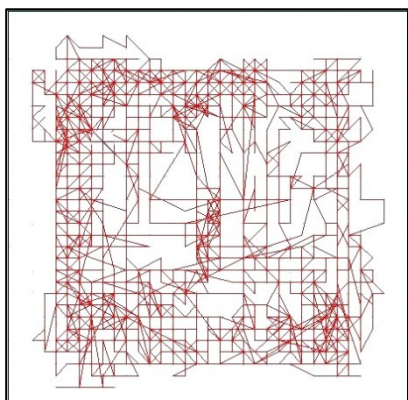**C**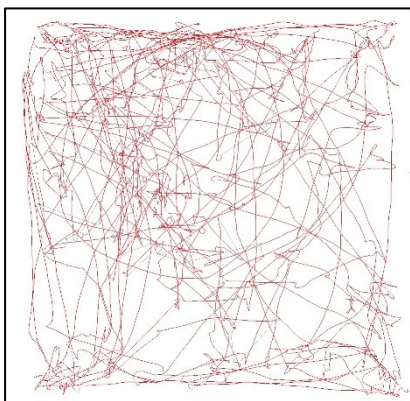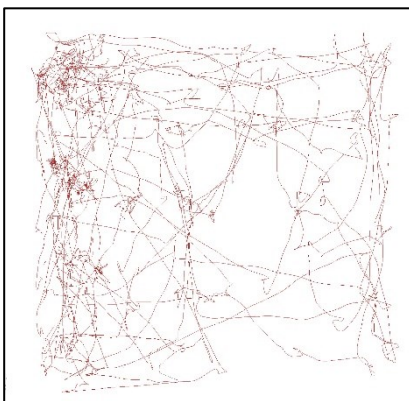**D**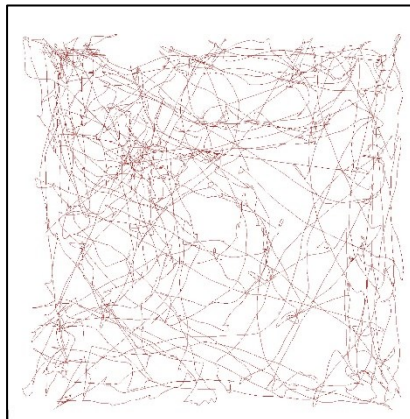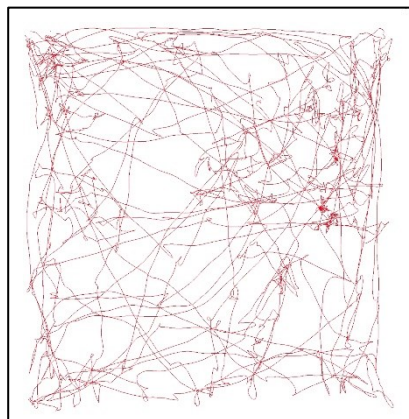

Supplementary Figure 2

Supplement: Supplementary file 6 — Supplementary Figure 2 [file 41398_2024_3014_MOESM6_ESM.pdf]
