# Supplementary material for: Differential effects of purified low molecular weight Poly(I:C) in the maternal immune activation model depend on the laboratory environment
Source: Transl Psychiatry. 2024 Jul 20;14:300. doi: 10.1038/s41398-024-03014-7 (PMC11271296; doi:10.1038/s41398-024-03014-7)
Supplement: Supplementary file 5 — Supplementary Table 3 [file 41398_2024_3014_MOESM5_ESM.pdf]

Supplementary Table 3A. Results of statistical analysis for cytokine responses

Maternal Plasma		Lab		Treatment		Lab:Treatment	
		$F_{(1,33)}$	p value	$F_{(1,33)}$	p value	$F_{(1,33)}$	p value
**VIE/ZHR 10 mg**	IFN-γ	5.349	0.027	91.698	<0,001	8.836	0.005
	IL-10	2.689	0.111	54.602	<0,001	6.611	0.015
	IL-1β	2.016	0.165	57.683	<0,001	4.930	0.033
	IL-6	5.049	0.031	88.359	<0,001	7.230	0.011
	TNF-α	4.448	0.043	86.434	<0,001	6.936	0.013
	Sickness	0.037	0.848	33.758	<0,001	0.303	0.586
		$F_{(1,36)}$	p value	$F_{(1,36)}$	p value	$F_{(1,36)}$	p value
**VIE/ZHR 20 mg**	IFN-γ	2.199	0.147	35.905	<0,001	2.492	0.123
	IL-10	12.342	0.001	93.540	<0,001	16.032	<0,001
	IL-1β	2.940	0.095	33.197	<0,001	3.474	0.071
	IL-6	1.488	0.231	63.535	<0,001	1.615	0.212
	TNF-α	1.908	0.176	51.799	<0,001	2.200	0.147
	Sickness	0.682	0.414	47.153	<0,001	1.473	0.233
**Fetal Brains**		**Lab**		**Treatment**		**Lab:Treatment**	
		$F_{(1,33)}$	p value	$F_{(1,33)}$	p value	$F_{(1,33)}$	p value
**VIE/ZHR 10 mg**	IFN-γ	0.217	0.644	1.151	0.291	0.597	0.445
	IL-10	29.545	<0.001	0.438	0.513	0.088	0.768
	IL-1β	0.706	0.407	3.365	0.076	0.247	0.622
	IL-6	0.624	0.435	12.772	0.001	0.673	0.418
	TNF-α	0.119	0.733	6.562	0.015	0.286	0.596
		$F_{(1,36)}$	p value	$F_{(1,36)}$	p value	$F_{(1,36)}$	p value
**VIE/ZHR 20 mg**	IFN-γ	1.578	0.217	2.194	0.147	1.720	0.198
	IL-10	44.433	<0.001	0.586	0.449	0.852	0.362
	IL-1β	2.198	0.147	2.473	0.125	1.697	0.201
	IL-6	5.469	0.025	12.889	0.001	4.733	0.036
	TNF-α	2.139	0.152	6.863	0.013	2.107	0.155
