# Supplementary material for: Differential effects of purified low molecular weight Poly(I:C) in the maternal immune activation model depend on the laboratory environment
Source: Transl Psychiatry. 2024 Jul 20;14:300. doi: 10.1038/s41398-024-03014-7 (PMC11271296; doi:10.1038/s41398-024-03014-7)
Supplement: Supplementary file 7 — Supplementary Figure 2 Legend [file 41398_2024_3014_MOESM7_ESM.pdf]

Supplementary Figure 2. Representative tracks of the Vienna (A, B) and the Zurich (C,D) cohort. A and C depict tracks of mice of the Poly(I:C) group, while B and D show tracks of the control group. Within each group tracks of one female and one male are shown.
